# Supplementary material for: Are carbon emissions trading and green financial instruments synergistic? -Comprehensive quantitative research based on content analysis
Source: PLoS One. 2024 Mar 7;19(3):e0298601. doi: 10.1371/journal.pone.0298601 (PMC10919601; doi:10.1371/journal.pone.0298601)
Supplement: S1 Data — (ZIP) [file pone.0298601.s001.zip › Data/地级市Z_F工作报告.zip!/╜¡╦╒/╦▐╟¿╩╨/└┤╫╘╣1⁄2╓┌║┼í╛┬φ┐╦╩2╛▌═°í┐_2016.docx]

各位代表：

现在，我代表市人民政府向大会作工作报告，请予审议，并请市政协各位委员和其他列席同志提出意见。

“十二五”经济社会发展取得了显著成就

“十二五”时期是宿迁综合实力提升最快、转型发展进展最大、人民群众得到实惠最多的时期之一。五年来，我们在省委、省政府和市委的正确领导下，在市人大、市政协的监督支持下，认真贯彻党的十八大、十八届三中四中五中全会和习近平总书记系列重要讲话精神，牢牢把握“迈上新台阶、建设新宿迁”发展定位，统筹推进稳增长、促改革、调结构、惠民生、防风险等各方面工作，国民经济持续健康快速发展，各项社会事业取得明显进步，顺利完成了“十二五”规划确定的目标任务，全面小康建设迈出坚实步伐，创造了宿迁发展的又一个黄金期。

从数字看发展，五年来宿迁综合实力显著增强，发展速度保持领先，经济结构持续优化，顺利迈入工业化中期阶段。面对复杂多变的宏观经济形势，始终把发展作为第一要务，牢牢把握稳与进的平衡点，超前研判、精准施策、定向发力，实现了经济发展换挡不失速、量增质更优。经济总量大幅攀升。地区生产总值五年增长近一倍，达2110亿元；人均地区生产总值达7000美元，达到中上等收入国家（地区）水平。一般公共预算收入由89.6亿元增加到235.7亿元，连跨两个百亿台阶。固定资产投资增长近3倍，达1839亿元。社会消费品零售总额翻了一番，达626.6亿元。增速位居全省前列。省列统的16项主要经济指标中有15项年均增幅达两位数，普遍快于全省和苏北平均水平。经济结构不断优化。三次产业占比由2010年的17.6:45.0:37.4调整到12.3:48.5:39.2，二三产业占比提高5.3个百分点，呈现协调并进的良好局面；工业化率突破40%，实现了从工业化初期向中期的跨越。县域经济整体提升。沭阳县连续四年跻身“全国百强县”，泗阳县入选全国县域经济竞争力百强县，泗洪县、宿豫区、宿城区综合实力明显增强。

从变化看产业，五年来宿迁工业制造业加速集聚升级，服务业质态逐步提升，农业高效规模发展，现代产业体系特色更加鲜明。大力实施产业强市战略，坚持年年都是工业突破年、年年都是项目推进年，强化产业集聚、催生产业集群、优化产业布局，促进产业由小变大、由大变强，产业转型升级取得重大进展。工业经济量质齐升。深入实施“511”工程，推进“三服务三促进”活动，规上工业增加值翻了近两番、达994.2亿元。市域“4+4”产业产值占比超过七成，县区“2+1”产业产值均占据半壁江山。

智能家电、绿色建材集聚成势，双双获批省级先进制造业基地。产业结构呈现“两升两降”，新兴产业和高新技术产业产值占比分别达25.1%和19.8%，提升了4.6个、13.2个百分点；高耗能行业产值增速下降6.4个百分点，单位工业增加值能耗下降23%。投资结构呈现“两快一慢”，设备投资、技改投资增速双双快于工业投资，“两高一资”行业投资增速减慢。产品结构呈现“两个提升”，智能冰箱、液晶电视、制冷压缩机等成功下线，产品科技含量和附加值进一步提升。企业质态不断优化，规上工业企业达2474户，产值过亿元企业翻了两番、达689户；主板上市和场外挂牌企业从2户增加到16户，一批在全省有地位、全国有影响的领军型企业崭露头角。新型服务业态加速成长。旅游业蓬勃兴起，项王故里、三台山森林公园等一批龙头型景区建成运营，去年以来一举创成4个国家4A级景区、1个省级旅游度假区，总数分别达9个和3个；2015年接待游客突破1600万人次，旅游收入达175.6亿元，均为“十一五”总和的1.5倍，实现了从短缺型旅游发展地区向重要旅游节点城市的历史性跨越。电子商务异军突起，中国“互联网+创业”大会成功举办，列为省跨境贸易电子商务试点城市，沭阳县、宿豫区获批国家级电商示范县（区），中国宿迁电子商务产业园区创成国家电子商务示范基地，中国淘宝镇、淘宝村数量分别占全省的1/3、1/5，快递业务量和业务收入均居全国第17位，电子商务交易额年均增长85.4%、达460亿元，“互联网+”已成为宿迁人创业就业的新时尚、增收致富的新渠道。金融服务体系更加健全，民生银行、太平洋保险、华泰证券等金融机构相继入驻，各类金融机构达128家，翻了近一番；存贷款余额分别达1819.8亿元、1696.7亿元，双双净增1000亿元。现代农业稳步提升。加强“1+4”特色农业产业体系建设，粮食生产实现“十二连丰”，四大特色产业产值比重提升至80.2%。设施农业、设施渔业分别达140.5万亩、25.4万亩，净增60.9万亩和16.1万亩；农业适度规模经营面积达303.5万亩，比重超过一半；农业机械化水平提高了9个百分点、达81%，农业生产方式加快从粗放型向集约型转变。农民参加专业合作社比重达65%，提高了26个百分点；家庭农场达5270个，居全省首位；家庭农场集群发展模式、“一村一品一店”建设经验全省推广，农业经营主体正在由单一向多元转变。这些变化不仅仅是量的积累，更标志着宿迁正在由农业资源大市向农业强市迈进。创新驱动显著增强。产业技术研究院从无到有、发展到10家，大中型工业企业基本建有研发机构，规上高新技术企业研发机构建有率达100%，省级创新型试点县区实现全覆盖；人才资源加速集聚，累计引进国家“千人计划”专家63人，入选省“双创人才”121人，人才总量突破40万人，宿迁日益成为各类人才成就事业、实现价值的热土；全社会研发经费支出占GDP比重预计达1.5%、增长了一倍多，科技进步贡献率提高了9.3个百分点、达51%，区域发展动力正由要素驱动加快向创新驱动转变。

从成果看开放，五年来宿迁项目招引成效斐然，载体平台升档进位，对外渠道加快拓展，开放发展呈现新的局面。牢固树立“四海”意识，坚持打开思路、拓宽视野，主动融入“一带一路”、江苏沿海开发等重大战略，友好交流合作与开放型经济发展实现互动并进。招商引资成果丰硕。创新“三位一体”招商机制，巩固传统区域，深耕港台地区，可功科技、格力电器等一批投资10亿元以上的大项目成功落户，累计引进亿元以上开工项目866个、竣工项目585个，分别是“十一五”的3.3倍、5.5倍。抢抓国家新一轮扩大开放机遇，以德国为重点的对欧招商局面全面打开，BGW、真维特高压、革创新型绿色建材等一批国际知名企业相继落户，实际到账外资累计达22.3亿美元，是“十一五”的3.7倍。外贸外经快速增长。有贸易往来的国家和地区突破200个、净增33个，进出口总额翻了一番、达26亿美元。新设境外企业29户，协议投资年均增长27%。载体建设提档升级。按照“五有三转”要求，大力推进开发区“二次创业”，宿迁及沭阳开发区成功创建国家级开发区，宿豫开发区获批省级高新区；6家共建园区综合考评持续位居全省第一方阵，苏宿工业园区连续六年名列第一。综保区、运河宿迁港建设稳步推进，海关检验检疫实现全国通关一体化。友城合作不断拓展。与新西兰因弗卡吉尔市、德国诺伊维德市缔结友好城市，友城数增至5个；与意大利摩德纳市、加拿大坎贝尔河市签署友好合作备忘录，与德国埃尔福特教育培训中心开展职教合作，宿迁国际“朋友圈”越来越大，对外交流合作层次越来越高。

从评价看城市，五年来宿迁个性特色愈加凸显，城市品牌全面打响，城乡面貌展露新姿，区域知名度和美誉度大幅提高。坚持多规融合、统筹兼顾，积极推进以人为核心的城镇化，全面加快城乡协调发展。全市城镇化率达55%、提高6.7个百分点，中心城市建成区扩大到85平方公里、人口增加到75万。城市发展广受赞誉。荣获中国人居环境奖城市、国家卫生城市、国家节水型城市、全国绿化模范城市、省优秀管理城市等称号，国家环保模范城市创建取得阶段性成果，文明城市直接晋升全国提名城市。根据美国米尔肯研究所发布的“中国最佳表现城市报告”和英国经济学人智库发布的“2015年中国新兴城市报告”，宿迁分别位列全国中小城市第四名和地级市第五名。自2012年起连续四年被台湾电电公会评为“值得推荐城市”。城市品质明显提升。从“补欠账”的里子工程做起，累计铺设雨污水管网650公里，实现雨污分流面积127平方公里，城市排水“动脉”逐步打通，城区基本实现“雨停路干”。从“强功能”的公共设施建起，新建改造环城西路、黄河南路等一批城市干道，市区“十一纵十二横”主干路网结构基本形成，城市通达能力显著增强；改造老旧小区20个、惠及市区近十分之一人口，完成市区213个小区物业整治提升，长期困扰居民的小区管理失序问题得到有效解决；加快城市商圈改造升级，金鹰购物中心、宝龙城市广场、苏宁广场等一批城市综合体开业运营，群众购物需求得到更高层次满足；深入实施中心城市民生六项工程，推进“两场一街一中心”建设，新建改造停车场24个、标准化菜市场17个、特色餐饮街区6个、社区服务中心和综合服务体8个；新增公共停车泊位3.58万个，建成遮阳棚、跳蚤市场等一批便民设施，城区公共厕所全部达到三类以上水冲式公厕标准且免费开放，让市民在细微处感受到城市的便利和人文关怀。从“塑个性”的特色绿化抓起，加快“四个城市”建设，累计实施438项园林绿化项目，打造了环城北路、千岛湖路、剧院路等一批林荫大道、彩色景观道和落叶景观路，建成了古黄河雄壮河湾公园、九鼎公园等一批生态公园，鸣凤漙、下相公园等一批精品游园加快推进，形成了花香迎宾、绿树绕城、庭院缤纷的城市个性特色。中心城市新（改）建绿地4.36万亩，绿地率、绿化覆盖率分别达39.5%、42.6%，人均公园绿地面积由12.14平方米提升到14平方米，“园林城市群”创建走在全省前列。宿迁既拥有了中小城市的舒适度，也具备了大城市的功能性，日益成为一座宜居宜业的城市。城市管理文明有序。成立市城管委，调整优化中心城市规划建设管理体制，城市环境综合整治三年规划任务全面完成，数字化城管实现区域全覆盖。订立“宿迁规矩”，常态化开展店外经营、违规遛犬等专项整治，市区“大花轿”全面禁行，广场舞噪音得到有效控制。培养“宿迁习惯”，以整治闯红灯和开展礼让斑马线活动为突破口，持续加大交通秩序整顿力度，强化宣传教育和严管重罚，推动“宿迁规矩”内化为“宿迁习惯”，促进了社会良好秩序的建立和巩固，交通文明指数全省第一。涵养宿迁文明，制定《宿迁文明20条》，通过“小习惯”唤起市民的文明自觉，文明有礼成为宿迁人的新特质。坚持市县联动，推动中心城市建设管理标准和导则向三县延伸，县城建设品位和档次得到大幅提升。泗阳县创成全国文明县城、国家卫生县城、国家园林县城，沭阳县、泗洪县通过国家卫生县城创建技术评估，宿迁在苏北率先实现省级文明城市全覆盖。农村面貌持续改善。大力开展“美好宿迁建设行动”，新（改）建农村公路3100公里、桥梁375座，新增农村无害化卫生户厕27.2万座，乡镇垃圾转运设施实现全覆盖，创建省三星级康居乡村48个、省“美丽乡村”建设示范村14个，建成一批乡里中心和公租农庄，农村田园风貌、地方特色愈加凸显。重大基础设施加快推进。通用机场完成选址，徐宿淮盐铁路正式开工，宿淮铁路、宿新高速、245省道等建成通车，古泊河船闸、成子河船闸等建成通航；完成分淮入沂整治、滞洪区安全建设等治淮骨干工程，建成“全省第三、苏北首个”全光网城市，基本形成以500千伏双泗变为电源支撑、220千伏中心城区和三县环网为骨干的供电网架。重大基础设施持续完善，既满足了宿迁发展的现实需要，也为长远发展打下了坚实基础。

从治理看生态，五年来宿迁空气质量不断改善，水体环境明显好转，绿地绿量逐年增加，生态宿迁的金字招牌擦得更亮。围绕打造“美丽宿迁”升级版，大力实施生态立市战略，抓源头重整治建制度，守住蓝天碧水，建好绿色家园，实现了经济发展与环境改善的良性互动。大气和水体整治成效明显。坚持从产生污染物的主体抓起，从污染物排放的源头治起，推进扬尘、工业废气等专项整治行动，累计淘汰黄标车14423辆、拆除燃煤锅炉632台，秸秆禁烧和综合利用水平稳居全省前列；全面开展“一企一管”“一企一测”“一企一治”，有效遏制了化工园区有机废气和无组织恶臭气体污染；市区环境空气质量优良率比2013年提升了5.6个百分点，PM2.5年平均浓度下降17.6%，超额完成省定目标任务。根据国家和省统一部署，全面取缔洪泽湖、骆马湖非法采砂，湖区生态得到有效改善，十多年不见的银鱼鱼汛再现骆马湖；按照“五全”治理思路，把水污染防治和城市功能完善结合起来，对市区古黄河、马陵河、西民便河等16条河道逐条排查整治，基本消除黑臭现象，重点河道变为城市水景，成了市民观光休闲的好去处。植树造林和生态修复扎实推进。以五大廊道建设为载体，持续开展植树造林，植树7688万株，新增林地23.4万亩，林木覆盖率提高到30.12%、位居全省第二，用全省1/12的国土创造了全省1/8的森林。创成国家级生态乡镇16个、省级34个。严守耕地保护和生态保护红线，生态红线考核跃居全省第四。生态基础工程加快建设。建成投运污水处理厂18座，日污水处理能力达63万吨，分别是“十一五”末的1.8倍和2.5倍。光大垃圾焚烧发电厂、有机生物处置中心、危险废物填埋场等先后建成，生活垃圾无害化处理率从38.7%提高至100%。体制机制不断健全。编制生态红线区域保护规划、骆马湖和洪泽湖生态环境保护总体规划等一批指导性规划。出台“1+5”文件，率先建立行政执法与司法联动的生态保护体制，构建了较为完善的生态保护制度体系。认真落实节能减排各项举措，坚决淘汰和改造落后产能，在经济持续增长的情况下，主要污染物排放强度逐年下降，化学需氧量、二氧化硫等指标超额完成省下达的“十二五”减排任务。

从制度看改革，五年来宿迁不等不靠率先探索，制度创新成果丰硕，施政行为科学规范，发展活力进一步释放。着眼治理能力和治理体系现代化，把改革贯穿于经济社会发展全局，把制度创新贯穿于改革始终，突出经济体制、农业农村、社会事业等重点领域，制定出台了近70项重要制度，用制度巩固改革、深化改革，确保改革红利最大化。行政管理制度更加合理。按照“减权去利、公开制衡”的理念，着眼行政管理行为全过程，构建“1+10”制度体系，推进行政审批制度、资格资质去行政化、非行政许可事项清理“三部曲”改革，全面建立市县两级权力清单和责任清单管理制度，进一步规范了政府“有形之手”。围绕“放”，制定深化行政审批制度改革总体方案、工商登记制度改革实施方案等一系列制度文件，核减审批事项、优化审批流程、创新审批机制，率先开展“四证（照）一体”“三证合一”“一证一号”等改革，行政审批事项由331项减至87项，成为全国行政审批效率最优的地级市之一；出台“两资”去行政化改革工作意见，在全国率先开展“两资”改革，569项资格资质项目仅保留53项、放开率达90.7%，进一步减少了对市场和社会主体的束缚。改革以来，全市年均新增私营企业16422户，其中公司制企业8698户、增长达122%。围绕“退”，研究制定推进政企分开、政社分开的一系列制度，推动市属国资平台退出竞争性领域，实施中介机构改革，减少不公平竞争和权力寻租，维护市场公平。围绕“接”，出台社会组织培育办法、政社联建工作意见，推行社会组织“五放五减十鼓励”登记举措，加大社会组织引培力度，社会组织总数达5726个，是“十一五”末的4倍。围绕“管”，制定行政审批制度改革监管体系建设工作导则，建立联动监管平台，构建社会信用体系基本框架，实现对市场主体的过程性监管，社会信用体系建设一跃进入全省第一方阵，成为全国首批创建国家社会信用体系建设示范城市。要素配置机制更加高效。围绕发挥市场对资源配置的决定性作用，制定公共资源市场化配置管理办法、产业发展基金管理办法、政府购买服务实施方案，推行创新券制度、地票交易制度、水气热合同交易制度和PPP模式等，促进了资源要素合理高效配置。社会事业服务机制更加健全。围绕构建“五大体系”，在14个乡镇开展卫生计生服务体制综合改革试点，在48家民营乡镇医院实施基本药物制度试点，基层医疗卫生服务水平进一步提升。出台深化民办教育改革与发展意见等文件，健全有利于民办教育发展的公共政策体系，保障民办教育持续健康发展。农村产权制度更加完善。在全省率先实施村级土地股份合作社、农村承包土地经营权抵押贷款和涉农贷款保证保险试点改革，县级农村产权交易平台实现全覆盖。119个乡（镇、街道、社区）、580余万亩农村土地基本实现“确实权、颁铁证”，比省定计划提前了一年。宿迁改革得到方方面面高度关注，央视多次聚焦报道，“两资”去行政化改革得到中央领导肯定，对全国改革产生了积极影响，国家层面陆续取消一批资格资质认证事项，以及组织机构代码和雷评收费，让市场主体大大受益。宿迁被省委主要领导赞誉为“改革发力最大、效果最好的地方”。

从获得感看民生，五年来宿迁公共服务体系日益健全，民生保障网越织越密，百姓生活丰富多彩，群众的幸福感和满意度越来越高。始终把人民群众对美好生活的向往作为奋斗目标，持续加大民生投入，推进民生幸福工程，群众分享到更多改革发展成果。省最新“六大体系”监测报告显示，我市住房保障体系水平指数、社会保障体系发展指数、社会养老服务满意度指数三项指标位居全省第一。在人民论坛测评中心发布的江苏省社会公平度测评中，宿迁位列全省第三、苏北第一。居民的收入更高了。大力弘扬创业文化，新（改）建创业载体173.4万平方米，新增创业13.8万人，越来越多的群众走上创业之路。加快公共就业服务体系建设，培训各类群体45.66万人，新增城镇就业23.97万人，转移农村劳动力26.3万人，城镇登记失业率实现“五连降”，工资性收入占比超过一半、成为居民增收的主渠道。城乡居民收入分别达22272元和12868元，比2010年净增9515元和5893元。得到的保障更全了。城镇职工“五险”扩面122.3万人次，城乡低保标准提高到490元/月和370元/月，城乡居民养老保险统筹并轨，新农合政策范围住院补偿比例提高到75%，大病保险实现全覆盖，各类困难群体保障水平大幅提升。完成棚户区改造14万户，基本建成各类保障性住房13万套，居民住房条件有效改善。搭建“12349”居家养老服务平台，千名老人拥有床位数达45.2张、居全省前列。享受的服务更优了。全市1310个村卫生室和112个社区卫生服务站全部达到标准化要求，实现基本药物制度村居全覆盖，群众小病不出村（居），就医负担大为减轻；市第一人民医院建成投用，二级以上医疗机构由21所增加到32所，三级医院由1所增加到4所，群众享受的优质医疗资源越来越多、越来越好。省优质幼儿园实现乡镇全覆盖，高水平通过全国义务教育发展基本均衡县（区）创建国家督导认定，本科达线人数连续五年超万人，宿迁学院成功申本；新建加固校舍547万平方米，校园D级危房全面消除；“八校”建设扎实推进，城区中小学布局进一步优化，孩子们在家门口就能上到好学校。集中力量实施区域供水“通村达户”工程，铺设供水管网2万公里，解决了290万人饮水不安全问题，广大农村居民喝上了同城市居民一样的优质自来水。市区新（改）建港湾式公交站台420个，新开辟公交线路41条，新增加新能源公交车412辆、空调车比重超过90%，候车时间由原来半小时以上缩短到10分钟左右，去年乘坐公交车人次突破9000万；先后建设143个站点、投放3350辆公共自行车，累计借车近260万人次、日均达5300人次；镇村公交开通率达88%，所有行政村实现客运班车通达，城乡居民出行更加舒适便捷。困难群体脱贫步伐更快了。三大片区及淮沭河沿线扶贫开发、黄河故道地区农业综合开发扎实开展，99.5万农村低收入人口全部达到年收入4000元的脱贫标准，360个经济薄弱村基本达到新“八有”目标，完成省定“十二五”扶贫任务。我市被列为国家扶贫改革试验区。精神文化生活更丰富了。市博物馆、图书馆、档案馆建成使用，文化建设工程综合指数跃居苏北首位，公共文化设施网络覆盖率位居全省前列；成功举办书画作品晋省展、晋京展，入展中国书法兰亭奖、全国美展等重要专业奖项人次和作品数量创新高，6部文艺作品获省第九届“五个一”工程奖；龙王庙行宫和中运河段入选大运河世界文化遗产，跻身全国文化城市百强榜。十八届省运会金牌数、奖牌数和总分数均实现翻番，宿迁籍运动员在伦敦残奥会、仁川亚残会披金斩银，奖牌数创历史新高；全民健身运动广泛开展，市体育中心和全民健身中心投入使用，十分钟健身圈初步构建；骆马湖国际生态四项赛广获好评，载入高校精品教材。“网上宿迁”名列全国政府网站绩效评估前三强，“宿迁之声”微博微信位居全国非省会地级市政务新媒体综合影响力榜首，“速新闻”成为全省最具影响力的地方新媒体。平安创建、综合治理、信访稳定工作成效明显，安全生产工作连续十年荣获省目标考核优秀奖，公众安全感排名全省前列。民防、物价、气象、地震、食药品安全、宗教、侨务、人民武装和双拥、档案、党史、地方志等工作实现了新进展，妇女、儿童、青少年、老龄、红十字、慈善等事业取得了新进步。

各位代表，在推进经济社会发展过程中，我们严格落实中央八项规定、省委十项规定和市委实施意见，扎实开展党的群众路线教育实践活动和“三严三实”专题教育，加强自身建设，转变政府职能，政府的执行力和公信力显著增强。大力提升服务效能，完善公开承诺、首问负责等制度，实行重大项目挂钩帮扶机制，加强便民方舟和政务服务“云平台”建设，畅通12345政府服务热线，创设“鼎鼎有民”网站、网络问政和《您知道吗》栏目，每月开展“星期三政民互动”活动；严厉查处不作为、慢作为行为，深化“群众不满意”事项整治，加大行政问责力度，政务服务环境进一步优化。深入推进依法行政，严格落实行政执法责任制，加强政府信息公开，建立新型政府法律顾问制度，制定（修订）市政府常务会议事规则、“三重一大”事项集体决策制度；自觉接受市人大及其常委会的法律监督和工作监督、市政协及社会各界的民主监督。全面加强廉政建设，深入开展专项整治，撤销各类领导小组和协调机构42个，清退办公用房2287间、4.89万平方米，“三公”经费下降15.4%；公车改革取得积极进展，取消的车辆全部封存到位；切实加强政风行风建设和审计监督，形成了风清气正的政风新常态。

回顾五年工作，经济社会发展取得了显著成就，积累了一些有益的经验：一是坚持遵循规律、锐意改革。始终遵从社会发展规律、经济运行规律和市场规律，自觉依规律决策、按规律办事，找准政府角色定位，注重把握政府与市场关系，厘清双方行动边界，并恪守这一边界，充分发挥市场对资源配置的决定性作用，更好地发挥政府作用，不断激发市场和社会主体活力，最大限度调动各方面积极性。二是坚持尽责有为、勇于担当。始终保持干事创业、奋发有为的精神状态，时刻保持迎难而上、攻坚克难的昂扬斗志，逆境面前不退缩，矛盾面前不回避，复杂疑难问题得到较好地统筹协调解决，集访和非访保持全省最低位，社会总体和谐稳定。三是坚持文明引领、善治善为。注重改进和完善社会治理方式，坚持以人文理念塑造城市文明，以规则建设促进公序良俗，公平公正有力地行使管理和服务责任，营造向上向善的社会秩序和社会氛围。四是坚持循法以动、依法行政。按照“法无授权不可为”“法定职责必须为”的原则，主动将行政行为纳入法治框架内考量，通过制度建设和考核导向强化依法行政的自觉性，做到不偏航、不越线、不逾矩。五是坚持尊重民意、凝聚共识。始终把民意作为最大的执政资源，畅通民意表达渠道，广泛倾听群众心声，让目标制定和项目推进的过程，成为集中民智、凝聚民力的过程。六是坚持系统谋划、层层推进。做决策通盘考虑、科学设计，想明白再干，想好了快干，确保各项工作善始善终、善做善成；抓落实从具体细节入手、从关键步骤发力，环环相扣、一抓到底，不达目的不罢休。

各位代表，“十二五”在宿迁发展史上谱写了辉煌篇章。这些成绩的取得，是全市人民团结拼搏的结果，是社会各界共同努力的结果。在此，我代表市人民政府，向奋战在各行各业的全体劳动者，向人大代表、政协委员、各民主党派、工商联和无党派人士、各人民团体，向离退休老同志，向中央和省驻宿单位以及人民解放军、武警官兵、公安干警，向参与宿迁建设的海内外客商、高层次人才、广大援宿干部，以及所有关心支持宿迁发展的各级领导、各界人士，致以崇高的敬意和衷心的感谢！

在肯定成绩的同时，我们也清醒地认识到发展中还存在不少困难和问题。主要是：经济下行压力加大，部分指标增速回落；经济发展不充分，总量仍然偏小，增长遇到瓶颈；实体经济困难加重，产业质态有待提升，转型升级任重道远；招商引资力度加大但成效不够突出，经济发展动力和潜力不足；城乡统筹任务艰巨，中心城市首位度有所提升但仍不明显，“三农”发展面临诸多难题；公共服务体系不够完善，民生工作尚有不少差距；政府职能转变有待加快，新常态下组织经济工作方式亟需改进，等等。对此，我们一定高度重视，采取有效措施，认真加以解决。

以全面建设小康社会引领“十三五”发展

各位代表，“十三五”时期是我市全面建成小康社会的决胜阶段。根据市委关于制定国民经济和社会发展第十三个五年规划的建议，市政府制定了“十三五”规划纲要（草案），提请大会审议。按照纲要（草案），“十三五”时期全市经济社会发展指导思想是：高举中国特色社会主义伟大旗帜，全面贯彻党的十八大和十八届三中四中五中全会精神，以马克思列宁主义、毛泽东思想、邓小平理论、“三个代表”重要思想、科学发展观为指导，深入贯彻习近平总书记系列重要讲话特别是视察江苏重要讲话精神，紧紧围绕全面建成小康社会、全面深化改革、全面依法治国、全面从严治党的战略布局，坚持发展是第一要务，坚持以工兴市第一方略，坚持稳中求进，坚持以提高发展质量和效益为中心，加快形成引领经济发展新常态的体制机制和发展方式，以“三抓三促”为工作主线，大力实施产业强市、城乡协调、民生优先、生态立市、创新驱动五大发展战略，统筹推进经济建设、政治建设、文化建设、社会建设、生态文明建设和党的建设，全力推动经济社会转型发展、绿色发展、创新发展，着力打造改革创新城市、青春活力城市、生态宜居城市，谱写好中华民族伟大复兴中国梦的宿迁篇章。主要奋斗目标是：实现“两个翻番”“两个确保”，即到2020年，地区生产总值和城乡居民收入比2010年翻一番，确保人均地区生产总值达到全国平均水平，确保不拖全省全面建成小康社会后腿。

在具体工作中，我们将围绕确立的目标任务，集中力量实施好五大发展战略。实施产业强市战略，推动经济转型发展。加快产业集聚升级，招大培强“双新一特”产业，提高一产发展水平，拉动三产快速发展。2020年，全市二三产业比重达90%，四大特色产业、四大新兴产业规上企业主营业务收入突破4800亿元。实施城乡协调战略，推进新型城镇化。推动中心城市和三个县城协调发展，稳步发展特色城镇，加快建设美丽村庄，着力构建具有宿迁特色的新型城镇化体系。2020年，全市常住人口城镇化率达到60%，中心城市和县城辐射带动能力不断增强。实施民生优先战略，提升社会保障水平。深入实施民生实事项目，持续增加居民收入，完善城乡社保体系。2020年，现行标准下农村贫困人口全部脱贫，人民群众生活质量、健康水平、居住条件明显提升。实施生态立市战略，走可持续发展之路。发展生态经济、绿色经济、循环经济、低碳经济，加快建设资源节约型、环境友好型社会，着力打造国家生态文明建设示范市。2020年，单位GDP能耗下降率完成省政府下达目标，空气质量良好以上天数达到70%。实施创新驱动战略，增强核心竞争优势。推动经济发展由要素驱动向创新驱动转变，强化大众创业万众创新，活跃市场新主体，不断培育和催生经济社会发展新引擎。2020年，社会研发经费支出占地区生产总值比重达到2%，成功创建国家创新型城市。

各位代表，实现“十三五”发展目标，是一项光荣而艰巨的任务。我们要准确把握战略机遇期内涵的深刻变化，主动适应新形势、新要求，有效应对各种风险挑战，切实担当起建成全面小康社会的历史使命，把“十三五”的宏伟蓝图变为美好现实！

努力为“十三五”开好局起好步

2016年是“十三五”的开局之年，也是宿迁建市二十周年。总的来看，今年还将继续面临复杂严峻的宏观经济形势，对于可能出现的困难应有充分的估计和足够的准备。我们将认真贯彻中央、省有关会议和市委四届六次全会精神，牢固树立“五大发展理念”，全面推进“五大战略”，坚持以“三抓三促”为工作主线，坚持稳中求进工作总基调，着力加强结构性改革，加快去产能、去库存、去杠杆、降成本、补短板，扩大有效供给，提高全要素生产率，加快培育发展新动能，全力推动经济社会持续健康发展，确保全面完成年度各项目标任务。全市经济发展的主要预期目标是：地区生产总值增长10%左右，一般公共预算收入增长12%左右，固定资产投资增长19%，社会消费品零售总额增长11%，全体居民人均可支配收入增长10%左右，节能减排完成年度目标任务。重点抓好七个方面工作：

（一）加快转型升级，增强经济发展活力。坚持产业强市战略不动摇，瞄准新型工业化方向，突出集聚升级，坚持量质齐抓，聚合力量做大做强做优主导产业。

推动“4+4”产业加速集聚。聚焦发展重点。按照市域“4+4”、县区“2+1”产业定位，围绕夯实基础、转型升级、高端引领三个层次，推进170个新型工业化项目建设，构建发展能级合理接续的产业“梯次”结构。做大产业规模。发挥传统产业在吸纳就业、提供税收等方面不可替代的优势，沉下心来做实做精，不断提高产品附加值和盈利能力。加大新兴产业规划引导和政策支持力度，推进产业规模化、布局集聚化，年内“4+4”产业规上企业分别实现主营业务收入2600亿元、400亿元。强化创新驱动。推动创新资源向企业集聚，加快产学研合作和科技成果转化，年内工业企业省级以上研发机构突破200家，技术交易服务中心建成投运。深化全国质量强市示范城市创建。加强高层次人才招引，年内引进国家“千人计划”和入选省“双创人才”各20名以上。

推动实体经济健康运行。以“511”工程助推企业转型。深入开展“四大行动”，年内完成千万元技改项目300户以上、兼并重组企业50户，新增股改上市企业7户、开票销售收入超2000万元企业300户。以“三服务三促进”减轻企业负担。认真落实“开展降低实体经济企业成本行动”的要求，围绕审批流程优化、正税清费、低价要素供给，压降企业“时间成本”“财务成本”“生产成本”，在为企业减负上打出“组合拳”。以产品升级提高市场供给水平。支持企业开展创新设计和品牌创建，引导企业与工业设计等公司合作开发新产品，研制新标准，提高产品市场占有率，年内创建中国驰名商标2个、省名牌产品12个以上。以定制经济促进供需优化。加快发展定制经济，依托京东“中国特产宿迁馆”等平台优势，鼓励企业更多运用定制理念扩大生产，促进消费与生产无缝对接，年内超过10%的规上工业企业拓展定制生产业务。

推动新增长点发展壮大。培育先导型产业。按照符合科技革新方向、绿色环保理念、人的个性化需求的要求，谋划推进一批先导型产业，抢抓发展先机，年内增材制造产业“一院三中心”建成投运，石墨烯涂层新材料加快产业化。做强电子商务产业。以创建国家电子商务示范城市为抓手，完善“一区多点”功能，引导电商新业态和知名电商企业加速聚集，推动电商同实体经济融合发展，更好更多地销售本地产品，带动更多的家庭就业创业，年内全市电商交易额突破700亿元，规上工业企业电商应用率提高到60%以上。提升旅游产业。实施旅游业发展新三年行动计划，重点推进三台山森林公园二期配套项目招商、项王故里景区三期规划建设，以及骆马湖-三台山国家级旅游度假区、洪泽湖国家级生态旅游示范区创建，打造乡村特色旅游点，全年接待游客达1850万人次，旅游收入突破200亿元。发展健康养老产业。加强“医养融合”体系建设，鼓励医疗机构开展医养结合型的养老服务。加快三台山森林公园健康养生区建设，打造国内知名的健康养老示范基地。

（二）强化“四海”意识，增强对外开放活力。积极融入国家和省对外开放的总体布局，把触角伸向“四海”，把能力拓展到“四海”，加快全方位开放步伐，汇聚更多资源要素。

集聚开放资源。深耕重点区域。巩固长三角、珠三角、港台等重点区域招商，以友城为支点主攻欧洲、兼顾日韩，组织好经贸洽谈会、绿色建材博览会和各类专题投资环境说明会，年内新开工亿元以上项目100个。创新招商模式。加强与知名产业研究院、专业咨询公司等合作，开展展会招商、网络招商等，提高重大项目招引精准率和成功率。优化招商机制。完善“三位一体”招商模式，制定优化招商引资机制方案，调动各类主体招商积极性。

拓展合作渠道。借力国家政策。主动融入“一带一路”、长江经济带等国家战略，抓好在手项目的对接和推进，电子商务、健康养老等产业纳入省重点产业发展规划，并加快工作进度，打造全省有位次、全国创品牌的特色产业基地；宿连航道等重大工程项目纳入省专项规划，尽早开工建设宿连运河先导段陆运河工程。推动友城建设。依托国际友城和友好交流城市，加强经济贸易、文体教育、科技人才等合作。积极涵养海外优质资源，为我市开拓新友城牵线搭桥。

加强载体建设。围绕建设特色型、生态型、集约型、创新型园区目标，按照“五有三转”要求，推动宿迁及沭阳两个国家级开发区实现更高层次发展，支持泗阳、泗洪及宿城开发区创建国家级开发区，支持宿迁高新区创建国家级高新区。进一步丰富合作共建内涵，把共建园区建成产业转移集聚区、外向突破引领区。推进综保区创建工作，宿迁二类水路口岸力争尽早获批。

优化营商环境。打造一流政策环境。抓住国家放松投资限制的机遇，逐步清理和废除妨碍统一市场的各种规定和做法，破除市场壁垒和地方保护，营造宽松的市场经营和投资环境，鼓励和支持各种所有制企业创新发展。打造一流法治环境。注重从制度、规则层面维护企业合法利益，加大对企业产权以及知识产权侵害行为的查处力度，用透明的法治环境稳定预期，给企业家以定心丸，充分发挥他们对经济发展的重要推动作用。打造一流服务环境。开展“新常态下营商环境建设提升年”活动，以更加优质的政务服务为企业保驾护航。加快构建同国际贸易和投资规则相适应的制度体系，通过营造规则的优势、政策的优势和服务的优势，促进国内外优质资源加速向宿迁集聚。

（三）突出内涵提升，增强中心城市活力。坚持人民城市为人民，认识、尊重、顺应城市发展规律，牢固树立“精明增长”“紧凑集约”理念，围绕“生态、精致、时尚”定位，致力在统筹上下功夫、重点上求突破、个性上做文章，着力提高城市发展持续性、宜居性，进一步彰显活力，提升城市竞争力。

以营建创新激发活力。探索实施“策创营”前置模式，切实提高城市项目建设运营的成功率。上半年完成国内外知名策划、创意和运营机构名录梳理及导则编制工作。开展新盛街等历史街区和特色街区项目营建，推动老城区改造与文化塑造相融合，留住城市特有的地域环境、文化特色、建筑风格等“基因”。

以人气集聚增添活力。繁荣城市商圈。推进中心城市商圈能级的合理分工，加快万达广场、西湖路商业街改造等项目建设，形成一级商圈与各区二级商圈互为补充的商业格局。推动房地产业转型升级。根据不同群体需求，细分市场、细分题材，推出差异化的住宅产品，引导房地产业差别竞争、健康发展。深入实施“房六条”政策，严格控制新土地出让，征收拆迁全部实行货币化安置、保障性住房全面实行货币化补助，打通供需通道，持续化解房地产库存。打造活力载体。鼓励企业和市民通过各种方式参与城市建设管理，实现城市共建共管、共建共享。围绕激发城市业态活力、人的创作活力、发展空间活力，建设城市活力区，在经营模式、业态生成、城市设计、色彩运用等方面充分发挥市民的主观能动性，让人的创造力成为增强城市活力的重要源泉。

以环境优化提升活力。完善公共设施。持续推进小餐饮集中疏导区、社区邻里中心等设施建设，深化“两场一街一中心”成果，提升城市综合服务功能。增强通达能力。实施城市道路更新计划，拓宽狭窄路面，疏通人行道、盲道和斑马线，打通“断头路”，完善城市路网体系。做好宿连高速、通用机场等重大交通项目前期工作，推进宿扬高速建设，运河宿迁港二期实现交工试运行。加快徐宿淮盐铁路和综合客运枢纽建设，完成宁宿徐高速公路宿迁主线收费站南移工程，实施好上海路快速化改造，打通和综合客运枢纽联系，形成以徐宿淮盐铁路宿迁站为核心的综合客运枢纽集疏运体系。提升管理水平。以全国文明城市创建为抓手，紧扣城市管理和服务，加强城市精细化管理，持续开展闯红灯、违规遛犬等整治行动，清理店外店、占道经营等违规行为，深入推行《宿迁文明20条》，打造良好的城市秩序和文明风尚。

以个性打造彰显活力。做足“绿秀”文章。围绕“四个城市”建设，按照节约型园林建设理念，高质量完成绿廊花廊、林荫道路等七大类、118项景观项目。进一步提升现有游园绿地功能，加强绿化管护，增设便民设施，打造配套完善的开敞公共绿地空间。做足“水韵”文章。推进骆马湖滨湖、中运河风光带等河湖沿线，以及氽园、青年公园等城市生态“水泡”建设，实现景观与生态深度融合。做足“山美”文章。加快三台山森林公园三期工程建设，高起点规划连山通湖走廊，建成“山、水、城”深度融合的城市景观带。做足“时尚”文章。编制时尚体育产业发展规划，创设一批时尚体育赛事。制定《民宿区专项规划》，发展都市农业、汽车旅馆等项目，构建时尚休闲的旅游特色。

（四）坚持协调并进，增强“三农”发展活力。把握“三农”发展新特点和新趋势，进一步发展规模农业，改善镇村面貌，深化扶贫开发，不断增强农业农村发展内生动力。

发展现代农业。打造特色农业板块。围绕“1+4”特色农业产业体系，全面落实粮食安全责任制，加快发展四大特色产业，构建“一企多社大园区”。推进“一村一品一店”。鼓励和支持龙头企业、专业合作社等主体开发符合网购需求的特色农产品，年内“一村一品一店”标准村（居）达60%以上。创新农业经营模式。培育各类新型经营主体，鼓励生产经营者大联盟，促进家庭农场集群建设，推进家庭、集体、合作社、企业等各类经营主体融合发展。

建设特色镇村。打造特色乡镇。筛选一批基础较好的乡镇，在建筑、人文、绿化等风格上提升辨识度，形成鲜明的个性。建设美丽村庄。按照康居村、美丽村、整洁村标准建设重点村、特色村和一般自然村，年内新增省“美丽乡村”建设示范项目10个。推进集中居住。完善集中居住区配套设施，建设乡里中心和公租农庄，促进农民生产方式、生活方式与社会管理方式同步转变。

深化脱贫攻坚。夯实精准措施。做好低收入人口和经济薄弱村建档立卡工作，做到对象精准；实施一户一策、一村一策，做到对策精准；落实机关企事业单位“挂村包户”脱贫责任制，做到“对子”精准；将扶贫开发与发展实体经济进行全方位对接，做到对接精准。做好“兜底”保障。整合各类救助资源，分类实施救助式、兜底式扶贫，确保完成年度扶贫开发任务。注重社会参与。开展政府购买服务试点，鼓励和引导社会组织承接政府扶贫项目，形成全社会共同参与扶贫开发的新局面。

（五）致力生态建设，增强绿色发展活力。实施生态文明建设三年行动计划，强化治理和保护，推动经济社会绿色发展。

筑牢生态安全屏障。开展绿化造林。实施五大林业重点工程，年内营造成片林3万亩。围绕五年更新改造115万亩雌株杨树、逐步消除杨絮污染的目标，深化“杨树更新改造年”活动，年内完成13.37万亩改造任务。构建生态廊道。推进环洪泽湖成子河船闸周边景观、环骆马湖公路景观、环城路绿化景观“三环”和运河景观带、古黄河风光带、民便河景观带、六塘河景观带“四带”建设，打造区域性大型绿色生态屏障。加强生态修复。实施林田河湖生态保护和修复工程，提升自然生态系统稳定性和生态服务功能，年内完成骆马湖4000亩围网养殖清理。

铁腕推进环境整治。开展废旧物资非法回收加工整治。按照“彻底禁、禁彻底”要求，对耿车、三棵树等地非法从事废旧物资回收加工的行为，一律予以取缔。开展城市河道整治。坚持截污与清淤并举，深化马陵河、古黄河、西民便河、中运河治理，年内马陵河完成整治，中运河完成周边环境整治和引导迁移等工作。开展空气异味整治。围绕化工、酿酒等重点行业，根治造成空气污染的各种顽疾，有效解决市区异味扰民问题。开展燃煤整治。全面淘汰供热管网、天然气管网覆盖范围内的燃煤锅炉，年内完成整治任务。

完善生态基础设施。推进污水处理设施建设。加快导流工程和城镇污水处理厂扩容提质工程建设，加大化工、印染、电镀等重点行业清洁化改造力度。推进垃圾收储设施建设。增设垃圾中转站，完善餐厨垃圾收运处置体系，扩大建筑垃圾资源化利用。加强农业废弃物和农业面源污染整治，推广畜禽养殖等农业废弃物综合利用技术。推进生态工程建设。探索以“DBO”模式，开展生态化污水处理试点，促进污水处理相关产业集聚。以部分住宅小区项目为试点，加快城市分布式能源建设。

（六）深化改革创新，增强社会主体活力。围绕推动供给侧结构性改革，进一步突出问题导向、发展取向、民生指向，加大重点领域和关键环节的改革力度，努力在体制、机制和技术等方面求突破，保持宿迁改革的先发优势。

深化行政管理体制改革。推动破除体制束缚类改革。开展多评合一、“互联网+”电子商务领域审批、工程建设“三联合”、设计施工一体化等改革试点，为市场主体提供更多便利。加快推进“两资”改革，扩大企业技能人才评价实施范围，完善市考试鉴定中心的公共服务功能。推动打破市场垄断类改革。强化四大国有投资平台功能和业务整合，做好机关事业单位下属企业剥离和分类处置。出台政府购买服务项目细化清单，并对指导目录进行动态调整。加快推进经营类检验检测机构与行政部门脱钩、转企改制，逐步走向市场。推动完善社会治理类改革。强化市社会组织服务中心辐射带动作用，加快县（区）社会组织孵化基地建设。以创建国家社会信用体系建设示范城市为抓手，全面加强社会信用体系建设，构建以信用监管为轴心的综合监管体系。

深化资源要素配置改革。调整优化供地机制。稳妥推进城乡建设用地增减挂钩、市域范围内村庄建设用地布局调整等改革试点工作；建立工业用地“亩产效益”评价体系，完善工业用地评估排序机制，探索差别化的供地机制。提升金融服务水平。继续大力支持企业开展直接融资，进一步提高新增直接融资比重。探索信用产品服务金融管理新模式，开展“厂商银”等供应链融资，年内新增贷款220亿元。推进要素价格改革。建立政府定价目录，明确政府定价范围。逐步推进涉房领域价费市场化改革，探索建立对民生热点行业的成本调查和发布机制。

深化社会事业改革。加强教育改革。建立学前教育基本公共服务制度，逐步构建多样化的学前教育服务供给体系。实施基础教育集团化办学，推进优质教育资源共享共用，实现教育质量整体提升。加强医疗卫生改革。进一步扩大乡镇基本药物制度试点范围，推进乡镇卫计服务体制综合改革，支持二级以上医院与下级医院建立医联体，提升基层医疗卫生服务能力，逐步满足群众多层次的健康服务需求。

深化农业农村改革。推进农村产权制度改革。完善县、乡两级农村产权交易平台，推动农村产权交易规范化运行，引导更多农村资产资源进场交易，全年交易总量不少于1000笔。推进农村土地制度改革。抓好农村土地承包经营权确权登记颁证扫尾及县级验收工作，上半年全面完成并顺利通过省级验收。加快村级土地股份合作社试点，进一步完善农村承包土地经营权和农村住房财产权抵押贷款机制，充分激发农村发展活力。

统筹推进其他改革。按照上级部署，结合宿迁实际，加大财税金融、科技体制、公共文化、司法体制、民主法制等方面改革力度，确保各项改革取得实效。

（七）立足便民惠民，增强民生保障活力。加大民生支出，扎实办好民生实事，增加公共服务供给，让宿迁的发展更有“温度”，让老百姓的幸福更有“质感”。

支持大众创业就业。做优公共服务平台。推动市、县、乡三级网络创业载体完善功能、提档升级，为创业者提供高效便捷服务。加快返乡创业园等各类创业载体建设，年内建成众创空间10个以上。开展创新创业活动。继续举办“互联网+创业”大会、“金种子”网络创业大赛等创业主题活动，引导大学生、退役士兵、农民工等重点群体走上创业之路。实施“家门口”就业工程。探索产业“下乡入户”发展与农村留守人员就近就业相结合的增收新路径，让更多低收入群体实现“家门口”就业。

促进社会事业发展。提升教育内涵。以教育现代化建设为统领，推进义务教育学校“改薄”、学前教育保基本、中小学校舍安全、中小学校“温暖行动”四项工程，加快中心城区中小学布局优化调整，努力办好家门口的每一所学校，年内70%以上义务教育学校达到省定现代化办学标准。优化卫计服务。实施村居卫生机构提质工程，加快市公卫中心、体检康复中心等项目建设，提高医疗卫生服务水平。落实一对夫妇可生育两个孩子政策。繁荣文体事业。完善公共文化设施网络，广泛开展文化惠民活动，提高文化供给能力。扶持文学、戏曲、书画、音乐等文艺事业发展，加快三台山文创基地建设，推动艺术精品创作再上新台阶。支持建设青年乐团和少年艺术团。开展全民阅读活动，打造书香宿迁。精心组织第四届国际生态四项赛等特色赛事，打响宿迁生态体育品牌。常态化做好“双拥”工作。

完善社会保障体系。优化社会保障。实施全民参保登记计划，推进社保扩面提标“幸福行动”，保障好各类困难群体基本生活。优化住房保障。加快棚户区和危旧片区改造，进一步改善群众居住条件。优化供水保障。逐步理顺供水体制，加强水源地管理，加快供水管网建设，年内全面完成区域供水工程。

强化社会综合治理。加强治安防控。推进社会治安防控体系建设，防范民生案件，保障人民群众安居乐业。抓好安全生产。严格落实安全生产责任，持续开展隐患排查治理和重点行业领域专项整治行动，确保不发生重特大事故。做好食品药品安全监管。改进信访维稳工作。实施社会稳定风险评估制度，推进阳光信访、责任信访和法治信访建设，维护改革发展稳定大局。

全面提升政府自身建设水平

各位代表，新形势、新任务对政府工作提出了更高要求。我们将巩固提升党的群众路线教育实践活动成果，自觉践行“三严三实”，加快转变政府职能，进一步增强政府执行力和公信力。

提升干事创业的本领。深入学习中国特色社会主义政治经济学理论，强化对供给侧结构性改革的研究和应用，增强新形势下组织经济工作的能力。提高“警商”，做到思想先人一步、行动快人一拍。完善政府领导集体学习制度、专家授课制度，健全公务员培训制度，提升政府工作人员业务素质和能力。

锤炼务实为民的作风。发扬苦干实干作风，把担当精神转化为推动发展的动力。组织开展作风建设专项整治，严厉查处有令不行、有禁不止和不作为、慢作为行为。深化政民互动，强化“四风”问题整改，解决“群众不满意”事项。以更严格的目标管理和监察问责，推动政务服务提速提质提效。

强化民主法治的理念。加强法治政府建设，认真落实重大决策公众参与、专家论证、风险评估、廉洁性审查、合法性审查和集体讨论决定等法定程序，提高工作法治化、科学化、民主化水平。主动接受人大及其常委会法律和工作监督，接受政协民主监督，高度重视社会舆论监督，把压力变成为群众谋利益的动力。

坚持廉洁自律的操守。严格落实新修订的《廉洁自律准则》和《纪律处分条例》，深入贯彻中央八项规定、省委十项规定和市委实施意见，不断强化“一岗双责”落实。推动公共资金、国有资产资源和领导干部履行经济责任情况审计全覆盖，进一步发挥好审计监督作用。坚持用制度管权、管事、管人，加强责任追究的制度化、程序化和常态化建设，从源头上预防腐败。

各位代表！宿迁加快发展的光荣使命，鼓舞我们砥砺奋进；全市人民的信任期盼，鞭策我们勇往直前。让我们在市委的坚强领导下，在市人大、市政协和社会各界的监督支持下，同心同德，务实奋进，为建成“强富美高”全面小康新宿迁而努力奋斗！
